# Apoptotic volume decrease (AVD) in A_549_ cells exposed to water-soluble fraction of particulate matter (PM_10_)

**DOI:** 10.3389/fphys.2023.1218687

**Published:** 2023-07-10

**Authors:** M. E. Giordano, G. Udayan, M. R. Guascito, A. R. De Bartolomeo, A. Carlino, M. Conte, D. Contini, M. G. Lionetto

**Affiliations:** ^1^ Department Biological and Environmental Sciences and Technologies (DiSTeBA), Salento University, Lecce, Italy; ^2^ Institute of Atmospheric Sciences and Climate, ISAC-CNR, Rome, Italy; ^3^ Institute of Atmospheric Sciences and Climate, ISAC-CNR, Lecce, Italy; ^4^ NBFC, National Biodiversity Future Center, Palermo, Italy

**Keywords:** AVD, particulate matter, apoptosis, A549 cells, oxidative stress, air pollution

## Abstract

Exposure to atmospheric particulate matter (PM) is recognized as a human health risk factor of great concern. The present work aimed to study the cellular mechanisms underlying cytotoxic effects of airborne particulate matter <10 µm in size (PM_10_), sampled in an urban background site from January to May 2020, on A549 cells. In particular, the study addressed if PM_10_ exposure can be a main factor in the induction of the Apoptotic Volume Decrease (AVD), which is one of the first events of apoptosis, and if the generation of intracellular oxidative stress can be involved in the PM_10_ induction of apoptosis in A549 cells. The cytotoxicity of PM_10_ samples was measured by MTT test on cells exposed for 24 h to the PM_10_ aqueous extracts, cell volume changes were monitored by morphometric analysis of the cells, apoptosis appearance was detected by annexin V and the induction of intracellular oxidative stress was evaluated by the ROS sensitive CM-H_2_DCFDA fluorescent probe. The results showed cytotoxic effects ascribable to apoptotic death in A549 cells exposed for 24 h to aqueous extracts of airborne winter PM_10_ samples characterized by high PM_10_ value and organic carbon content. The detected reduced cell viability in winter samples ranged from 55% to 100%. Normotonic cell volume reduction (ranging from about 60% to 30% cell volume decrease) after PM_10_ exposure was already detectable after the first 30 min clearly indicating the ability of PM_10_, mainly arising from biomass burning, to induce Apoptotic Volume Decrease (AVD) in A549 cells. AVD was prevented by the pre-treatment with 0.5 mM SITS indicating the activation of Cl^−^ efflux presumably through the activation of VRAC channels. The exposure of A549 cells to PM_10_ aqueous extracts was able to induce intracellular oxidative stress detected by using the ROS-sensitive probe CM-H_2_DCFDA. The PM_10_-induced oxidative stress was statistically significantly correlated with cell viability inhibition and with apoptotic cell shrinkage. It was already evident after 15 min exposure representing one of the first cellular effects caused by PM exposure. This result suggests the role of oxidative stress in the PM_10_ induction of AVD as one of the first steps in cytotoxicity.

## 1 Introduction

World Health Organization (WHO) recognizes that air pollution is a critical risk factor for noncommunicable diseases (NCDs) all over the world. It is estimated that air pollution is causing 24% of global adult deaths from heart disease, 25% from stroke, 43% from chronic obstructive pulmonary disease, and 29% from lung cancer. Air pollution is a major risk factor for pneumonia, being the leading cause of death in under 5 years aged children (WHO, 2019). In particular, the particulate component of air pollution poses a major risk to health. Atmospheric particulate matter (PM) is a complex mixture of components with great variability in their physical-chemical properties according to climatic, geographical, and source-specific variables (Amato et al., 2016; Chirizzi et al., 2017). The size of particles is directly linked to their potential for causing health problems. Small particles less than 10 μm in diameter pose the greatest problems because they can get deep into the respiratory tract, and some may even get into the bloodstream, in particular fine inhalable particles. According to WHO, exposure to PM has been identified as an important risk factor for mortality (WHO, 2019). The International Agency for Research on Cancer (IARC) has classified particulate matter from outdoor air pollution as carcinogenic to humans (IARC Group 1) (Loomis et al., 2013).

Although exposure to PM is recognized as a human health risk factor, the causal relationship between PM exposure and the genesis of pathological conditions and the underlying toxicological mechanisms are to date not completely understood. Several studies outlined the oxidative potential of PM (Chirizzi et al., 2017; Romano et al., 2020) and its capability to induce intracellular oxidative stress (Lionetto et al., 2019; Lionetto et al., 2021), as an important property for the outcome of adverse health effects (Cheng et al., 2016). In general, oxidative stress has been associated with cell homeostasis imbalance, mitochondrial damage, and apoptosis –(Yang et al., 2018; Zhang et al., 2018). Though numerous studies have focused on the cytotoxic effects of PM so far, several issues remain unclarified, particularly those related to the cell death pathways and underlying mechanisms (Chen et al., 2023).

Exposure to PM is known to be associated with apoptosis induction in several cell types including bone marrow (BM)-derived endothelial progenitor cells (Cui et al., 2015) through oxidative stress induction, human epithelial lung cells (L132) through activation of both TNF-α induced pathway and mitochondrial pathway (Dagher et al., 2006), mice bronchial epithelium cells via PI3K/AKT/mTOR signaling pathway (Han and Zhuang, 2021), GC-2spd cells by activation of RIPK1 apoptotic signaling pathway (Zhang et al., 2018), alveolar macrophages (Wei et al., 2021), and human cardiomyocytes (AC16 cell) (Yang et al., 2018) through mitochondria-mediated apoptosis pathway.

The apoptotic process is associated with a distinct set of molecular and cellular changes involving the cytoplasm, nucleus, and plasma membrane, which include cell shrinkage, formation of apoptotic bodies, chromatin condensation, and DNA degradation. The apoptotic cell shrinkage is a universal prominent feature of the cell under apoptosis and arises in two distinct phases: The first phase starts before cell fragmentation or formation of the apoptotic body, while the second phase is associated with cell fragmentation (Benson et al., 1996). The early phase, called Apoptotic Volume Decrease (AVD) (Maeno et al., 2000), is represented by an isotonic cell shrinkage that occurs early after apoptotic stimuli, before the activation of caspases, the release of cytochrome c from mitochondria and DNA fragmentation, and seems to be a prerequisite for apoptosis (Okada et al., 2001). In a variety of cell types, prevention of AVD inhibits subsequent apoptotic biochemical and morphological events, and cells are rescued from death (Maeno et al., 2000; Antico et al., 2023).

The cell volume reduction during apoptosis occurs under normotonic conditions, independent of changes in the osmolarity of the extracellular environment, and is the consequence of an exit of Cl^−^ and K^+^ from the cells (Bortner and Cidlowski, 2002; Lionetto et al., 2010; Poulsen et al., 2010; Antico et al., 2023) through the activation of specific channels. Concerning Cl^−^ exit from the cell, volume-regulated anion channels (VRAC) are considered the players in vertebrate cells (Maeno et al., 2000; D’Anglemont de Tassigny et al., 2004). These channels are formed by a hexameric assembly of members of the LRRC8 gene family and are ubiquitously expressed in all vertebrate cells being involved in cell volume homeostasis (Bertelli et al., 2021). The molecular mechanisms underlying their activation have not yet been completely understood. The proposed molecular mechanisms include low intracellular ionic strength, membrane unfolding, oxidation, phosphorylation, and G-protein coupling (Bertelli et al., 2021). It is known that VRAC activation is crucial to AVD happening and it occurs rapidly in a wide variety of cell types in both mitochondrion-mediated intrinsic, and death receptor-mediated extrinsic apoptosis (Maeno et al., 2000; Shimizu et al., 2004; Lee et al., 2007). Inhibition of these channels was found to prevent AVD and subsequent downstream apoptotic steps (Okada et al., 2006).

The present work aimed to study the mechanisms underlying cytotoxic effects of airborne particulate matter <10 µm in size (PM_10_) in A549 cells, used as a model, focusing on one of the earliest events of apoptosis, the Apoptotic Volume Decrease (AVD). A549 cells are representative of the human lung Alveolar Type II pneumocytes (Foster et al., 1998), and are being widely used as a cellular model for respiratory research and assessment of adverse effects of PM on human health (Yi et al., 2012; Wang et al., 2013). Type II cells are the only cells involved in surfactant secretion in the respiratory epithelium and their damage can affect the lung defense system against environmental stressors (Akella and Deshpande, 2013).

For the study, we used aqueous extracts of eight airborne PM_10_ samples, collected in an urban site (Aradeo, province of Lecce, Puglia, Italy) potentially influenced by the local urban activities, biomass burning, agricultural activities, and the nearby industrial activities during the period from 14-01-2020 to 28-05-2020. Guascito et al. (2023) described that the larger contribution of PM_10_ in the study site originated from biomass burning.

The choice of water-soluble extracts of sampled PM_10_ arises from the experimental need to reproduce experimental conditions similar to the physiological exposure at the level of respiratory epithelium, where the surface of the respiratory epithelial cells is covered by a thin fluid layer, in which PM_10_ dissolves.

To the best of our knowledge, this is the first work focusing on airborne PM and AVD induction and aims to contribute to improving the knowledge about the mechanisms underlying the effects of PM at the airway epithelium.

## 2 Materials and methods

### 2.1 Sampling campaign, PM_10_ gravimetric determination and chemical composition

The study was performed on eight PM_10_ samples chosen from the whole sampling campaign carried out from December to May 2020 in a site located in southeastern Italy (Aradeo, Puglia, Italy) already described in a previous work (Guascito et al., 2023). The sampling site was located in the center of the municipality of Aradeo (Lecce, Italy) (40◦07′47″ N; 18◦07′56″ E) with a population of about 10,000 inhabitants. The site is an urban background site potentially influenced by the local urban activities, biomass burning and agricultural activities, and the nearby industrial activities including a cement production plant located at about 7.2 km in the northeast direction. Daily PM_10_ samples (starting from midnight) were collected by an automatic low-volume sampler at 38.3 L·min^−1^ (Zambelli Explorer Plus) on PFTE filters (Whatman, 47 mm in diameter) located on the roof of the City Hall at about 14 m above the ground.

As widely described by Guascito et al. (2023), the larger contribution of PM_10_ in the study site originated from biomass burning.

Gravimetric determination of PM_10_ samples was done according to UNI EN 12341 (2014) by weighing the filters (three replicates before and after sampling), following stabilization for 48 h in a conditioned room (for details see Guascito et al., 2023). The weighing was performed using a microbalance Sartorious Cubis (model MSx6.6S, ±1 μg resolution). Quality control of gravimetric results was done using field blanks and periodic (once per week) control of the inlet flow rate of the samplers with external flow meters. Organic (OC) and elemental carbon (EC) were determined by a Sunset laboratory carbon analyser (Sunset Laboratory Inc., Tigard OR, United States) using thermo-optical transmittance (TOT) with the EUSAAR2 protocol (Cesari et al., 2018). The analyser was calibrated using a sucrose solution as an external standard (2.198 g/L in water, CPAchem Ltd., Bulgaria). Linear calibration had a slope of 0.97 (±0.01), a negligible intercept, (0.1 ± 0.2), and a determination coefficient *R* ^2^ = 0.99.

The chemical composition of PM_10_ sampled was determined via ICP-MS (PerkinElmer NexION 1000 and NexION 300x) for the main metals and ion chromatography (ICS1100, Thermo Scientific) for the water-soluble ions according to Guascito et al. (2023).

### 2.2 Cell viability measurement by MTT assay

Cell viability was evaluated by MTT test on A549 cells exposed for 24 h to the water-soluble fractions of PM_10_ extracted from the whole PFTE filter for each of the eight samples according to Lionetto et al. (2021). Extraction was carried out in 10 mL ultrapure water (Milli-Q) using an ultrasonic bath. Four cycles of sonication for a total of 80 min were performed and each cycle was followed by 1 min vortex agitation (according to Lionetto et al., 2021). Then, the extracts were filtered using PTFE (polytetrafluoroethylene) 0.45 μm pore syringe filters. The assay measures the metabolic activity of the cells as an indicator of cell viability, assessing the mitochondrial NAD(P)H-dependent oxidoreductase enzyme activity which reduces a yellow tetrazolium salt (3-(4,5-dimethylthiazol-2-yl)-2,5-diphenyltetrazolium bromide (MTT) 3-(4, 5-dimethylthiazol-2-yl)-2, 5-diphenyltetrazolium bromide to formazan that accumulates as crystals within healthy cells. These are dissolved with DMSO and the absorbance of the resulting colored solution is spectrophotometrically analyzed at 570 nm (Cytation 5, BioTek Instruments, Winooski, VT, United States). Six replicates per sample were carried out. The relative viability of the cells was calculated as follows:

$$Relative\;viability\;of\;cells\;\left(\%\right)=[\left(treated\;cells\;OD\right)/\left(untreated\;cells\;OD\right)]\;x\;100$$

### 2.3 Morphological analysis of the cells and cell volume change determination

A549 cells adherent to the bottom of a 96 multiwell were exposed to the PM_10_ aqueous extracts for 24 h and visualized by Cytation 5 cell imaging multimode reader (Agilent, Santa Clara, CA, United States) (observation objective: ×40). Cell volume changes were monitored by morphometric analysis of the cells and were expressed as a percentage of the cell area of 2-D cell images after PM_10_ exposure vs. the cell area of control cells (cells not exposed to the PM_10_ extracts) according to Giordano et al. (2020) and Lionetto et al. (2010). At least a minimum of 100 cells/field and 5 fields per well were analyzed.

### 2.4 Detection of apoptosis by annexin V

One of the earliest events of apoptosis is the translocation of the membrane phospholipid from the inner to the outer leaflet of the plasma membrane. Once exposed to the extracellular environment, binding sites on phosphatidylserine become available for Annexin V, a 36 kDa Ca^2+^-dependent phospholipid-binding protein that has a high affinity for the anionic phospholipid phosphatidylserine (Leventis and Grinstein, 2010).

A549 cells exposed to PM_10_ aqueous extracts for 24 h were incubated with 1 μg/mL annexin V (Alexa Fluor^®^ 488) for 15 min and viewed by Cytation 5 cell imaging multimode reader according to Gelles et al. (2019).

### 2.5 Intracellular oxidative stress detection assay and confocal visualization

The intracellular oxidative stress was assessed using the ROS-sensitive cell-permeant probe 5-(6)-Chloromethyl-2′,7′-dichlorodihydrofluorescein diacetate, acetyl ester (CMH_2_DCFDA) (Ex/Em: 492–495/517–527 nm) (Thermo Fisher Scientific, Waltham, MA, United States) according to Lionetto et al. (2021); Giordano et al. (2020). The probe, once in the intracellular compartment, loses its acetate group, which is cleaved by cellular esterases, and undergoes hydrolysis. The resulting DCFH carboxylate anion is trapped inside the cell and once oxidated by intracellular ROS produces the fluorescent product DCF (Ameziane-El-Hassani and Dupuy, 2013). Fluorescence intensity was measured using the Cytation 5 cell imaging multimode reader. The results were expressed as a fold increase in the fluorescence intensity compared to the negative control (untreated cells). More details of the methodology are reported in Giordano et al. (2020).

Cells charged with CM-H_2_DCFDA were also visualized by confical microscopy. Briefly, A549 cells were plated at a density of 1 × 10^5^ cells per mL into a chambered coverslip (IBIDI, Gräfelfing, Germany), incubated for 24 h for the adhesion of the cells to the bottom of the plate, then exposed for 24 h with the aqueous PM_10_ extracts and finally charged with CM-H_2_DCFDA as reported above. The cells were viewed using a ×100 NA plan apochromatic objective mounted on a NIKON TE300 inverted microscope coupled to a NIKON C1 confocal laser scanning unit (Nikon, Tokyo, Japan). The Argon 488-nm laser line was used. Unlabeled cells did not exhibit any detectable fluorescence under the conditions used. Images were acquired and analyzed by EZ-C1 NIKON software.

### 2.6 Statistics

Data are given as the mean ± S.E.M. The statistical significance was analyzed by one-way ANOVA, and Dunnett’s multiple comparison test.

## 3 Results

### 3.1 Effect of PM_10_ exposure on cell viability

The average values of PM_10_ concentration and carbon content of the 8 samples used in the present study are reported in Table 1. The sample set included six winter samples characterized by higher PM_10_ concentrations and two spring samples characterized by lower PM_10_ values in agreement with the typical seasonal PM_10_ concentration of the area (Cesari et a., 2018). On average the same trend was also observed for carbon content. Moreover, it must be considered that the sampling campaign was carried out in 2020, when Italy was subjected to a national lockdown and limitation to the movement of people because of the COVID-19 pandemic. The first six samples were collected before the lockdown period, while the other two May samples were collected just after the lockdown when the restart of activities was slow. Some previous evidence indicated that the lockdown has slightly reduced the PM_10_ concentration in the air at least in this region of Italy (Dinoi et al., 2021). The mean chemical composition of the PM_10_ in the study site is reported in Supplementary Table S1.

**TABLE 1:** Values of PM_10_ concentrations and carbon content (organic carbon OC and elemental carbon EC) of the eight samples used for the study.

Sampling data	PM_10_ (µg/m^3^)	OC (µg/m^3^)	EC (µg/m^3^)
14/1/2020	47.9 ± 2.4	29.6 ± 1.5	3.2 ± 0.2
18/1/2020	50.8 ± 2.5	21.8 ± 1.1	2.0 ± 0.1
21/1/2020	37.9 ± 1.9	17.8 ± 0.9	1.9 ± 0.1
23/1/2020	35.4 ± 1.8	4.6 ± 0.3	0.4 ± 0.1
11/2/2020	27.1 ± 1.3	4.7 ± 0.3	0.5 ± 0.1
5/3/2020	27.9 ± 1.4	11.6 ± 0.6	1.3 ± 0.1
9/5/2020	14.1 ± 0.7	3.3 ± 0.2	0.4 ± 0.1
28/5/2020	12.9 ± 0.6	2.6 ± 0.2	0.3 ± 0.1

**FIGURE 1:**
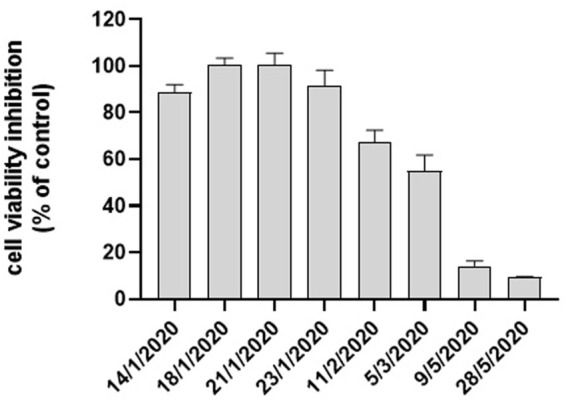
Cytotoxicity of PM_10_ expressed as % cell viability inhibition assessed by the MTT test on A549 cells exposed for 24 h to PM_10_ aqueous extracts.

Cell viability after 24 h exposure to water-soluble extract of the airborne PM_10_ samples was assessed by MTT assay on A549 cells. In Figure 1 the MTT results obtained on the eight PM_10_ samples chosen for the objective of the present study are reported. Inhibition of the mitochondrial NAD(P)H-dependent cellular oxidoreductase enzymatic activity after exposure of A549 cells for 24 h to the aqueous extracts of PM_10_ compared to control cells was observed in the sample data set in agreement with a previous study on the whole sampling campaign (Guascito et al., 2023). The entity of the inhibition varied from about 100% (detected in January samples) to values of about 9% recorded in May samples. The dose-dependence response of the assay for each aqueous extract was preliminarily checked by exposure of A549 cells to increasing dilutions of the same extract, as reported in our previous studies (Lionetto et al., 2019; Lionetto et al., 2021). On average, winter samples showed a higher cytotoxic potential as indicated by the high percentage reduction of cell viability, particularly in January and February, compared to the two May samples according to Guascito et al. (2023) in parallel to PM_10_ concentration.

### 3.2 Cell volume alteration and apoptosis detection in A549 cells exposed to PM_10_

To deepen the analysis of the mechanisms involved in PM_10_-induced cytotoxicity on A549 cells, cell morphology was analyzed on PM_10_ exposed cells in parallel to MTT assay using Cytation 5 cell imaging multimode reader on the same samples.

**FIGURE 2:**
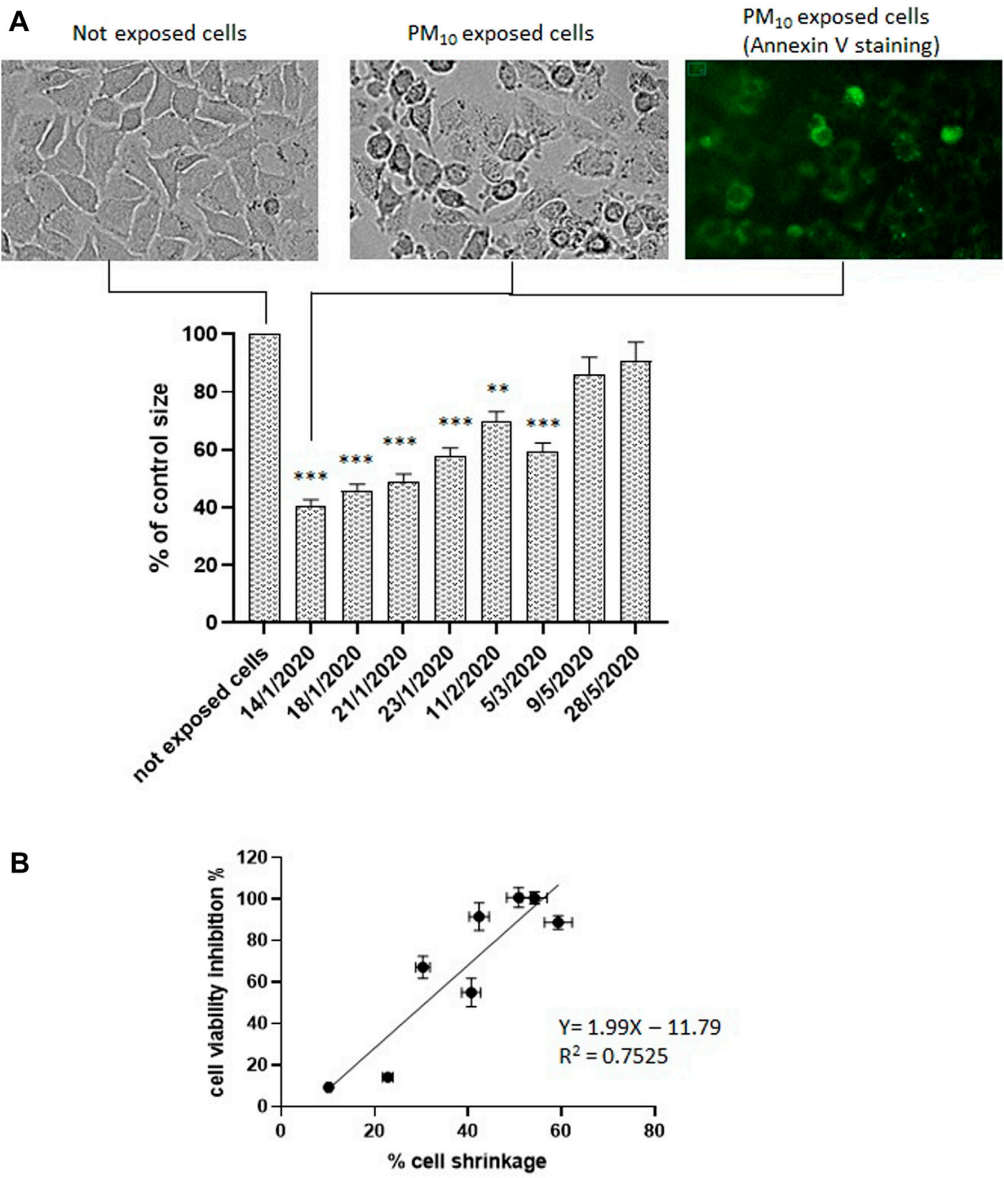
**(A, B)** Cell volume change in A549 cells exposed to the aqueous extracts of the 8 PM10 samples expressed as percentage cell size compared to not exposed cells (control). Representative brightfield image of not exposed cells; representative images of PM_10_ exposed cells (sample of 14-1-2020). brightfield and annexin V stained. **(B)** Correlation analysis between cell viability inhibition and % of cell shrinkage.

After 24 h exposure to PM_10_ aqueous extracts, A549 cells showed typical cell shrinkage detected as a percentage decrease of the cell area of 2-D images compared to not exposed (control) cells (Figure 2A). Cell shrinkage levels were in agreement with the NAD(P)H-dependent cellular oxidoreductase enzymatic activity reduction, as indicated by the correlation analysis (Figure 2B). Particularly high cell volume reduction, above 50%, was observed in the samples which showed the highest inhibition of mitochondrial NAD(P)H-dependent oxidoreductase activity. Cell shrinkage was also accompanied by other typical morphological signs of apoptosis such as cell rounding, surface roughness, blebs formation, and annexin V positivity, visualized by the delectable green contour of the cell plasma membrane of cells treated with 1 μg/mL annexin V (Alexa Fluor^®^ 488) following exposure to PM_10_ extract as shown in Figure 2A. This figure reports representative images obtained from the sample of 14-1-2020. Similar apoptotic positive evidence (cell rounding, surface roughness, blebs formation, and annexin V positivity) were obtained also for the other 5 winter samples but was not detectable for the two May samples (not shown).

### 3.3 Apoptotic volume decrease in A549 cells exposed to PM_10_ aqueous extracts

After detecting reduced vitality and apoptosis appearance in A549 cells following 24 h exposure to PM_10_ aqueous extracts, we tested the hypothesis of the possible involvement of PM_10_ exposure in the induction of the Apoptotic Volume Decrease (AVD), which is one of the first events of apoptosis, occurring early in the first 1–2 h (Chang et al., 2000; Maeno et al., 2000). Therefore, the time course of the volume changes was monitored in A549 cells during the first 2 h of exposure to PM_10_ aqueous extracts by time-lapse imaging of the cells every 30 min. The AVD analysis was focused on 4 representative samples, two January samples, characterized by the highest values of vitality reduction and a marked cell shrinkage after 24 h exposure, and the two samples of May, characterized by a reduction of the NAD(P)H-dependent cellular oxidoreductase activity below 15% and without apparent apoptotic signs. For each of the four filter extracts, three independent experiments were performed.

**FIGURE 3:**
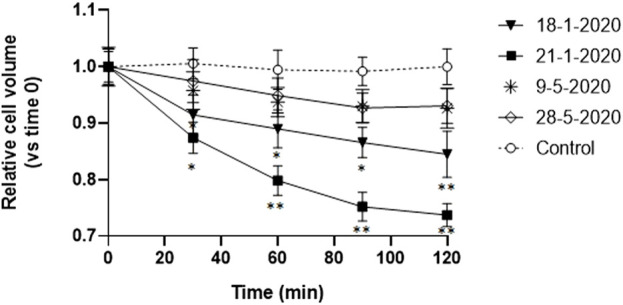
Time-course of cell volume change in A549 cells during the first 2 h of exposure to the aqueous extracts of 4 PM10 samples. Cell volume changes were expressed as percentage cell size compared to the size of the cells at time 0. *p < 0.05; **p < 0.01.

The cells exposed to the aqueous extracts of January 18 and 21 showed a significant reduction of cell volume compared to control cells already after 30 min exposure and the decrease continued in the 2 h observation reaching 16% and 27% respectively (Figure 3). If these results are compared with the overall apoptotic cell volume reduction observed after 24 h in the same extracts, the AVD response observed in the first 2 h corresponds to one-third and one-half respectively of the overall cell shrinkage produced.

On the other hand, the cells exposed to the other two spring extracts (9-5-2022 and 28-5-2022) did not show any significant cell size reduction compared to the control cells.

### 3.4 Effect of SITS in the PM_10_-induced apoptotic volume decrease

**FIGURE 4:**
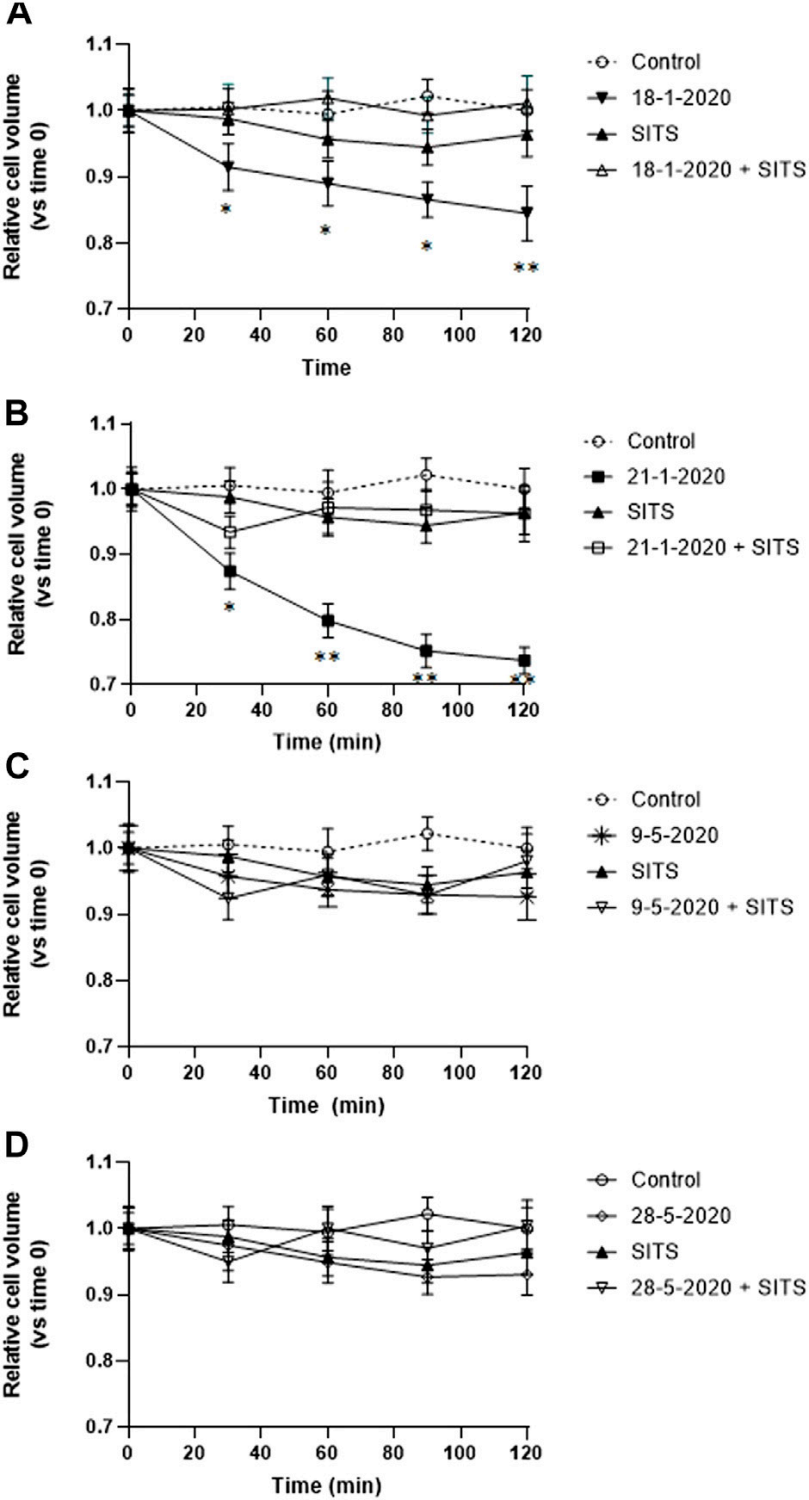
**(A–D)** Effect of SITS (0.5 mM) on the time-course of cell volume change in A549 cells during the first 2 h of exposure to the aqueous extracts each of the 4 PM10 samples. Cell volume changes were expressed as percentage cell size compared to the size of the cells at time 0. *p < 0.05; **p < 0.01.

AVD is known to be caused by the loss of K^+^ and Cl^−^ from the cell (Yu and Choi, 2000). Therefore, in order to investigate the nature of the PM_10_-induced early isotonic cell shrinkage, A549 cells were preincubated with 0.5 mM disulfonic stilbene derivative (4-Acetamido-40-isothiocyanato-stilbene-2,20-disulfonic acid), a known inhibitor of Cl^−^ channels (Kokubun et al., 1991) including the ubiquitously expressed volume-regulated anion channels (VRACs) (Maeno et al., 2000; Hoffman et al., 2015; Okada et al., 2021), which have been demonstrated to be involved in AVD in other cell types (Maeno et al., 2000). The cells were viewed by time-lapse microscopy. After (4-Acetamido-40-isothiocyanato-stilbene-2,20-disulfonic acid) preincubation, the cells were exposed to the PM_10_ aqueous extracts of 18-1-2020 and 21-1-2020 for 2 h and in this case, the PM-induced isotonic shrinkage was completely prevented (Figures 4A, B). On the other hand, (4-Acetamido-40-isothiocyanato-stilbene-2,20-disulfonic acid) alone was not able to produce any significant alteration in cell size. The other two extracts, not showing AVD, were also tested and no significant effect of (4-Acetamido-40-isothiocyanato-stilbene-2,20-disulfonic acid) treatment was observed (Figures 4C, D).

### 3.5 Oxidative stress induction in A549 cells exposed to PM_10_

It is known that reactive oxygen species and oxidative stress play a pivotal role in apoptosis induction in several cell types (Kannan and Jain, 2000; Redza-Dutordoir and Averill-Bates, 2016).

**FIGURE 5:**
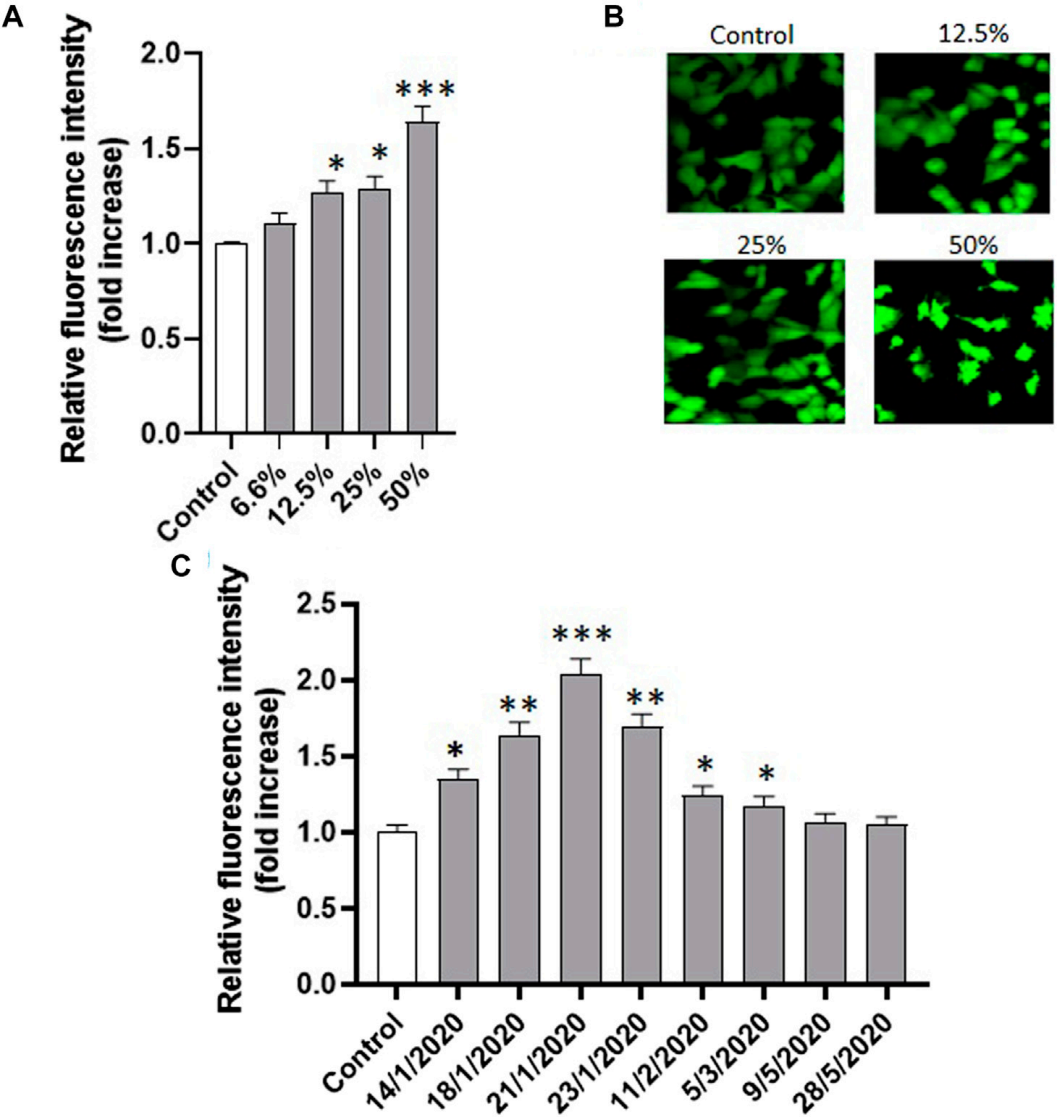
**(A)** Effect of the exposure (24 h) to increasing concentrations of the same PM_10_ extract (representative experiment on the sample of 21-1-2020) on the intracellular fluorescence intensity of A549 cells charged with the ROS-sensitive probe CM-H2DCFDA and the corresponding confocal images **(B)**; **(C)** Intracellular fluorescence of A549 cells exposed to the eight PM_10_ aqueous extracts for 24 h and charged with the ROS sensitive probe CM-H2DCFDA (extract dilution used for all the samples: 50%).

In order to test whether oxidative stress was involved in PM_10_-induced apoptosis in A549 cells, intracellular oxidative stress levels were measured in A549 exposed for 24 h to all the eight PM_10_ aqueous extracts of the study using the cell-permeable probe CM-H_2_DCFDA. Preliminarily, the cells were exposed to different dilutions of the same extract. Figure 5A shows the representative dose response for one of the tested samples (21-1-2021) revealing that PM_10_ was able to induce the generation of intracellular oxidative stress in a dose-dependent way. The confocal representative images of cells exposed to different dilutions of the PM_10_ extracts and then charged with the fluorescent probe are reported in Figure 5B. The intracellular fluorescence intensity of the exposed cells increases with the concentration of the PM_10_ aqueous extract. At the highest dilution tested the appearance of apoptotic blebs is clearly evident.

**FIGURE 6:**
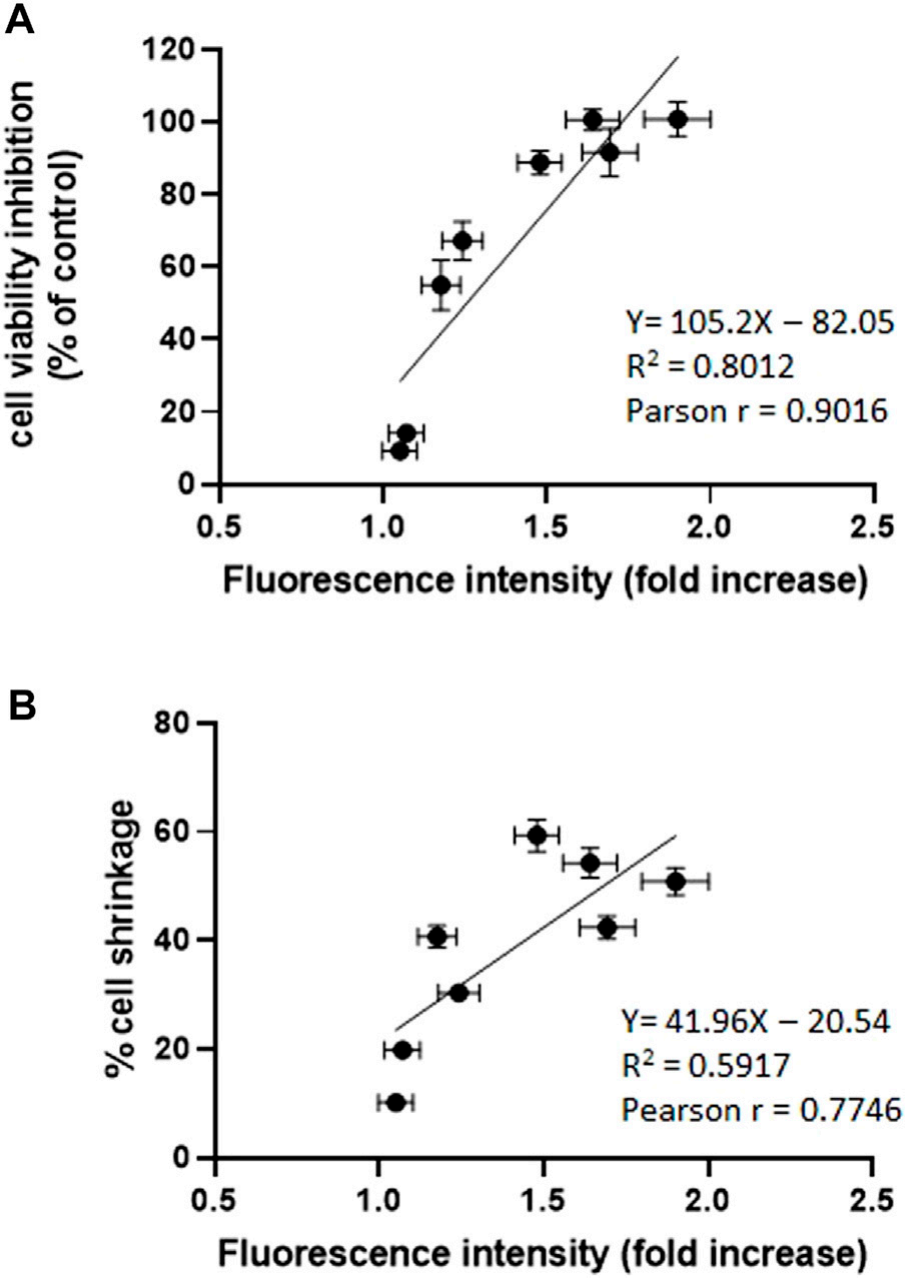
**(A)** Correlation analysis between cell viability inhibition and intracellular oxidative stress expressed as fold increase of the fluorescence intensity of the CM-H2DCFDA probe compared to control. **(B)** Correlation analysis between apoptotic cell shrinkage evaluated after 24 h and intracellular oxidative stress (see above) ***p < 0.001; **p < 0.01.

The analysis of the PM_10_-induced intracellular oxidative stress was applied to the eight extracts revealing a highly significant fluorescence increase compared to control in the six winter samples after 24 h, while no significant effect was observed in the two spring samples (Figure 5C). The oxidative stress data were statistically correlated with vitality inhibition data and cell shrinkage results (Figures 6A, B) suggesting that the generation of intracellular oxidative stress can be one of the main underlying mechanisms of the PM_10_ induction of apoptosis in A549 cells.

**FIGURE 7:**
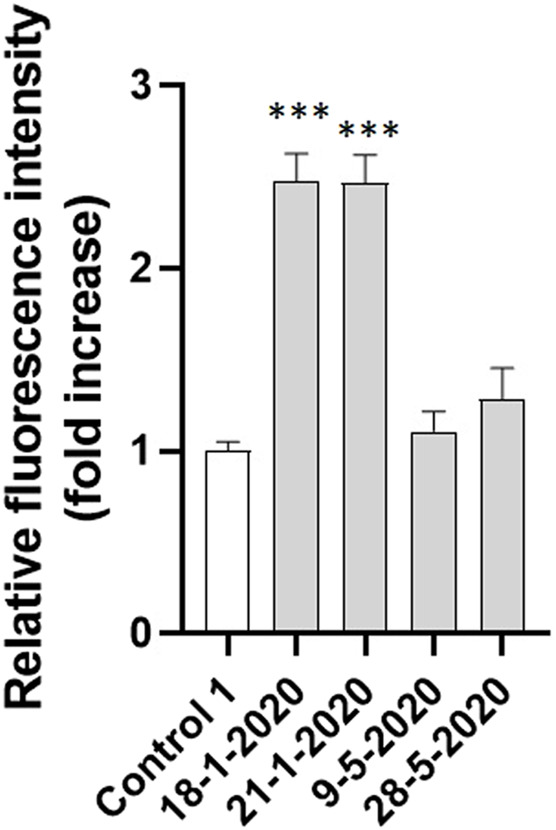
Intracellular fluorescence of A549 cells exposed to the four PM_10_ aqueous extracts for 15 min and charged with the ROS sensitive probe CM-H_2_DCFDA (extract dilution used for all the samples: 50%). The statistical analysis of data was performed by one way ANOVA and Dunnett’s multiple comparison test.

The investigation of the effect of PM_10_ exposure on intracellular oxidative stress induction was deepened with a short-term analysis of the intracellular fluorescence of A549 cells charged with CM-H_2_DCFDA and exposed for 15 min to the 4 aqueous PM_10_ extracts used for AVD analysis. As reported in Figure 7, the cells exposed to the aqueous extracts of 18-1-2020 and 21-1-2020 expressed a significantly increased fluorescence compared to control cells already after 15 min exposure, suggesting that the induction of oxidative stress by PM_10_ was an early event, timely correspondent with the AVD induction sustained by VRAC channels.

## 4 Discussion

The impact of air pollution on public health has become a great concern worldwide, in particular, PM is considered one of the main risk factors for human health (Loomies et al., 2013; WHO, 2019). Cell death has been used as a hallmark of cell injury induced by PM (Peixoto et al., 2017) representing a general toxic outcome since it results from the integration of the multiple toxic effects that PM can induce at the cellular level. Cell death has been recognized as one important underlying mechanism of the development or exacerbation of respiratory diseases, such as emphysema and chronic obstructive pulmonary diseases (Pleixoto et al., 2017).

The present work wants to contribute to the knowledge about the mechanisms underlying the cytotoxic effects of PM at the airway epithelium using A549 cells as a model and focuses on the induction of apoptotic volume decrease, one of the early events in the apoptotic process. The study was carried out on water extracts of airborne PM coming from an urban site in the South of Italy (Aradeo, province of Lecce, Apulia, Italy) whose dominant PM source was represented by biomass burning during the sampling period of the study as previously assessed by Guascito et al. (2023). The used samples included winter samples with high PM_10_ values, high carbon content values (mainly linked to biomass combustion, as previously assessed), and high cytotoxicity, and spring samples, collected in the post-COVID-lockdown period, characterized by low PM_10_ values, low carbon content values, and low or negligible cytotoxicity.

Cell death has a central role in homeostasis and it is also responsible for the onset of several pathological conditions. In recent years, various types of cell death, such as apoptosis, autophagy, necrosis, pyroptosis, ferroptosis, and cuproptosis, have been elucidated (Chen et al., 2023), and an increasing number of works have focused on the cellular death pathways related to PM exposure. Among the death cell types, apoptosis is the most widely investigated in PM-induced cytotoxicity and it has been related to the appearance of pulmonary fibrosis (Yang et al., 2020).

In our study, cytotoxic effects induced by PM_10_ ascribable to apoptotic death were observed in winter samples which are characterized by the higher PM_10_ and organic carbon content, as revealed by the typical apoptotic sign appearance such as cell volume reduction, cell rounding, surface roughness, blebs formation, and annexin V positivity. Apoptotic volume reduction was observed early, since a significant cell shrinkage was already detectable after the first 30 min exposure to the winter PM_10_ samples, while no significant changes in cell volume were observed in spring samples which did not show the typical sign of apoptosis. These results clearly indicate the ability of PM_10,_ mainly arising from biomass burning, to induce AVD in A549 cells. AVD was triggered by the activation of Cl^−^ efflux, since the pretreatment with SITS, a known inhibitor of Cl^−^ channels including the VRAC channels, completely inhibited the PM-induced activation of AVD. The pretreatment with SITS did not exert any effect on cell volume in control conditions suggesting that the channels responsible for the PM_10_-induced Cl^−^ efflux were not activated in basal conditions.

A549 cells are known to express the ubiquitous volume-regulated anion channels (VRAC) composed of members of the leucine-rich repeat-containing protein 8 family (LRRC8A-E) (He et al., 2010; Canella et al., 2017; Bach et al., 2018; Centeio et al., 2020). Normally, these channels are closed and underwent activation following hypotonic swelling playing a key role in the Regulatory Volume Decrease response which allows the cells to recover their volume through the release of osmolytes (mainly K^+^ and Cl^−^) followed by loss of osmotically obliged water. In addition, VRAC channels have been previously demonstrated to be activated in A549 cells in normotonicity under apoptosis-related stressful conditions such as carboplatin and ozone exposure (He et al., 2010; Canella et al., 2017; Bach et al., 2018) being responsible for the induction of AVD. In light of this knowledge, our results indicate the ability of biomass-burning-related PM_10_ to activate AVD in A549 cells through the activation of VRAC channels.

As regards the PM_10_ components able to induce the activation of VRAC channels, it is known that these channels are activated by ROS in various cell types (Jiao et al., 2006; Matsuda et al., 2010; Shen et al., 2014; Friard et al., 2021). PM is known to express an intrinsic oxidant-generating capacity that is related with the physical-chemical properties of the particles, such as their surface characteristics and their chemical composition, related to the pollutants adsorbed (such as metals, polycyclic aromatic hydrocarbons, quinones) expressed by the PM oxidative potential (Chirizzi et al., 2017; Carlino et al., 2023). Besides its intrinsic oxidant-generating capacity, PM also exhibits a cell-mediated oxidant-generating capacity once in the cells, including the activation of intracellular signaling pathways leading to the formation of ROS, the interference with the endogenous production of ROS at the mitochondrial level, the release of radical metabolites arising from the biotransformation of the chemical contaminants of PM (Ghio et al., 2012), and the inhibition of intracellular antioxidant defenses (Chirino et al., 2010). The two types of PM oxidant properties (endogenous and cell-mediated) are statistically correlated as previously demonstrated (Lionetto et al., 2021; Guascito et al., 2023).

In our study, we found that exposure of A549 to PM_10_ aqueous extracts was able to induce intracellular oxidative stress detected by the ROS-sensitive probe CM-H_2_DCFDA. The oxidative stress induction, expressed as a fold increase of the intracellular probe fluorescence, was statistically significantly correlated with cell viability inhibition and with apoptotic cell shrinkage. The oxidative stress induction was already evident after 15 min exposure suggesting that the appearance of PM_10_ induced AVD through the activity of VRAC channels is mediated by the PM_10_ induced intracellular oxidative stress, which in turn represents one of the first PM-induced cellular effects leading to cytotoxicity.

## 5 Conclusion

In conclusion, obtained results demonstrated for the first time that exposure to airborne PM_10_ aqueous extracts, mainly influenced by biomass burning, induces Apoptotic Volume Decrease in A549 cells. AVD was prevented by the pre-treatment with SITS suggesting the activation of Cl^−^ efflux presumably through the activation of VRAC channels. The exposure of A549 cells to PM_10_ aqueous extracts was able to induce intracellular oxidative stress detected by using the ROS-sensitive probe CM-H_2_DCFDA. The PM_10_ oxidative stress induction was statistically significantly correlated with cell viability inhibition and with apoptotic cell shrinkage. The oxidative stress induction was already evident after 15 min exposure representing one of the first cellular effects induced by PM exposure leading to cytotoxicity. Its early appearance suggests its role in the PM_10_ mediated AVD induction. This finding deserves to be in more detail evaluated in future works addressing the efficiency of the endogenous antioxidant system in PM treated A549 cells.

Although future studies are needed to better clarify important aspects of the research such as the signaling pathway accounting for AVD activation through VRAC opening and the role played by ROS in these pathways also in relation to the chemical composition of PM_10,_ the manuscript contributes to improving the knowledge about the cellular mechanisms responsible for the effects of PM at the airway epithelium.

## Data Availability

The raw data supporting the conclusion of this article will be made available by the authors, without undue reservation.
