# Supplementary material for: Apoptotic volume decrease (AVD) in A549 cells exposed to water-soluble fraction of particulate matter (PM10)
Source: Front Physiol. 2023 Jul 10;14:1218687. doi: 10.3389/fphys.2023.1218687 (PMC10364053; doi:10.3389/fphys.2023.1218687)
Supplement: Supplementary file 1 [file Table1.DOCX]

**Supplementary information**

**Apoptotic Volume Decrease (AVD) in A_549_ cells exposed to water-soluble fraction of Particulate Matter (PM_10_)**

Giordano M.E.^1^*, Udayan G.^1^, Guascito M.R.^1^, De Bartolomeo A.R.^1^, Carlino A.^1^, Conte M.^3^, Contini D.^2^, Lionetto M.G.^1*^

^1^Dept. Biological and Environmental Sciences and Technologies (DiSTeBA), Salento University, Lecce, Italy

^2^Institute of Atmospheric Sciences and Climate, ISAC-CNR, Str. Prv. Lecce-Monteroni km 1.2, 73100 Lecce, Italy

^3^Institute of Atmospheric Sciences and Climate, ISAC-CNR, Via Fosso del Cavaliere, 100 - 00133 Rome, Italy

Tecnologie, l'Energia e lo Sviluppo Economico Sostenibile (ENEA), Rome, 00123

Correspondence should be addressed to:

*Maria Giulia Lionetto

Dip.to di Scienze e Tecnologie Biologiche e Ambientali

Università del Salento

Via prov.le Lecce-Monteroni

73100 Lecce

Italy

Tel +39 0832 298668

Fax +39 0832 298626

e-mail: [giulia.lionetto@unisalento.it](mailto:giulia.lionetto@unisalento.it)

*Maria Elena Giordano

Dip.to di Scienze e Tecnologie Biologiche e Ambientali

Università del Salento

Via prov.le Lecce-Monteroni

73100 Lecce

Italy

Tel +39 0832 298668

Fax +39 0832 298626

e-mail: [elena.giordano@unisalento.it](mailto:elena.giordano@unisalento.it)

**Table S1.** Chemical composition of PM_10_ in the samples of the study site. Average and standard deviation (in parenthesis).

| **Species** | **µg m^-3^** | **Species** | **ng m^-3^** |
| --- | --- | --- | --- |
| SO_4_^2-^ | 2.09 (1.20) | V | 0.91 (0.62) |
| NO_3_^-^ | 3.68 (1.92) | As | 0.83 (0.66) |
| K^+^ | 0.43 (0.21) | Cd | 0.1 (0.06) |
| Cl^-^ | 2.34 (1.96) | Cr | 0.80 (0.65) |
| Br^-^ | 0.06 (0.06) | Mo | 0.24 (0.16) |
| NH_4_^+^ | 0.87 (0.55) | Ni | 9.2 (9.7) |
| Mg | 0.53 (0.34) | Pb | 2.9 (2.2) |
| Na | 1.45 (2.93) | Se | 0.4 (0.4) |
| Fe | 0.161 (0.124) | Sn | 1.0 (0.6) |
| Zn (*) | 0.025 (0.016) | Tl | 0.16 (0.10) |
| Mn (*) | 0.005 (0.002) | Co | 0.03 (0.06) |
| Cu (*) | 0.005 (0.003) | Sb | 1.22 (1.66) |
